# The Size Effect on the Phase Transition and Dielectric Properties of Poly(vinylidene Fluoride) Ferroelectric Polymers

**DOI:** 10.3390/polym17091286

**Published:** 2025-05-07

**Authors:** Xiaofang Zhao, Min Yu, Xining Zhang

**Affiliations:** School of Mathematics, Statistics and Mechanics, Beijing University of Technology, Beijing 100124, China; minyu@emails.bjut.edu.cn (M.Y.); zxning@emails.bjut.edu.cn (X.Z.)

**Keywords:** ferroelectric polymer, size effect, interfacial layer, phase transformation, dielectric properties

## Abstract

Multi-scale characterization techniques have been employed to analyze the size effect of microstructure on the phase transition behavior and dielectric properties of poly(vinylidene fluoride) (PVDF) films. The results show that oriented amorphous fraction layers are prone to form in the vicinity of the grain boundaries of nano-grained films, while the interfacial polarization and electrostriction effect play a major role. Polar nano-regions are prone to form in micro-grained films, and the maximum fraction of polar crystalline phase and maximal dielectric constant can be achieved due to the balance between the intrinsic effect and extrinsic effect of the material. On the contrary, the extrinsic effect corresponding to interfacial charges greatly influences the phase transition behavior between beta and alpha phases for coarse-grained PVDF films, while the dielectric properties are mainly influenced by the intrinsic electrostatic field and van der Waal interaction of the material. Hence, the dielectric behavior of nano-grained films can be adjusted by the copolymerization technique, that of micro-grained films can be adjusted by both the copolymerization technique and the controlling of microstructure morphology, and that of coarse-grained films can be adjusted by the doping technique.

## 1. Introduction

In recent years, increasing attention has been paid to ferroelectric polymers, as this kind of material has wide applications in new electromechanical and thermoelectrical transducers. Investigations about the dielectric, ferroelectric and pyroelectric properties of ferroelectric polymers have fascinated many researchers and play an important role in nanotechnology.

Polyvinylidene fluoride (PVDF) is one typical semi-crystalline material whose crystalline polymorphs have at least four phases, while the nonpolar α phase and polar β- and γ-phases make up the majority of the crystalline material [1]. The morphology of ferroelectric polymers is similar to polycrystalline structures with diffusional grain boundaries [2], and the mechanical constraint effect arising from the mismatch between crystal lattices of coexisting phases and the related depolarization effect corresponding to the local motion of molecular chains greatly influences the crystallinity of the materials [3]. Meanwhile, interfacial charges or head–head/tail–tail (HHTT) defects can accumulate at the interfaces between the crystalline phase and amorphous phase of ferroelectric polymers [4], which affect the dielectric permittivity and piezoelectric coefficient of the material. Hence, both the mechanical constraint and the interfacial charge distribution in the vicinity of the grain boundaries influence the mechanical and dielectric properties of the PVDF-based polymers by transforming the configuration of the molecular chains. In a recent work of the authors [5], PVDF films were manufactured with glass substrates, and it was found that the crystallinity and ferroelectric properties of PVDF films are greatly related to the grain size, while the extrinsic effect can be ascribed to the mechanical constraint and the distribution of HHTT defects in the vicinity of the grain boundaries. Note that the film thickness and grain size are correlated for dielectrics with a grain-like microstructure configuration [6], and the doping technique induces the existence of free charges in the ferroelectric polymers. Hence, for the PVDF films, the extrinsic effect should include both the mechanical constraint and the electrical field corresponding to free charges in the vicinity of the grain boundaries, and the size effect should be further analyzed by considering both the influences of the film surface and grain morphology.

As is well known, the microstructure and phase transformation of ferroelectric polymers are influenced by both extrinsic effects and intrinsic effects, where the extrinsic effect mainly induces variations in lattice spacing and the electrostatic field distribution and the intrinsic effect greatly relates to the van der Waals interactions among the molecular chains. Note that the grain boundary region of ferroelectric polymers consists of the oriented amorphous fraction (OAF) layers and isotropic amorphous fraction (IAF) layers [1,7]. Nevertheless, to the best of the authors’ knowledge, to date, researchers have paid little attention to the size effect of the microstructure morphology on the dielectric property and phase transition behavior of ferroelectric polymers.

In the present study, PVDF films with various grain sizes and film thicknesses were manufactured with silicon substrates. The microstructure configurations, phase transition and dielectric relaxation behavior of PVDF films were investigated to analyze the size effect of microstructure morphology on the phase transition behavior and dielectric properties of the material.

## 2. Materials and Methods

As described in the authors’ previous work [5], PVDF films were manufactured by the spin-coating technique, where silicon substrates of dimensions 10 × 10 mm^2^ were used. The spin-coating rate and annealing temperature adopted in the present experiment are listed in Table 1. And the microstructure, phase transition behavior, crystallinity, and lattice structure were measured by STEM (FE-STEM SU9000; Hitachi, Chiyoda, Japan), DSC (214 Polyma; Netzsch, Selb, Germany), XRD (D8 DISCOVER; Bruker, Billerica, MA, USA) and FT-IR (B420-PE (Spectrum II); Perkinelmer, Waltham, MA, USA) devices, respectively.

Note that, in the present study, the variation in dielectric constant and dielectric loss with temperature was measured with a dielectric analyzer (E4980A; Agilent, Santa Clara, CA, USA), while the scanning temperature range was set as 25–150 °C, and the magnitude of the alternating electric field and the scanning frequency range were set as 0.5 V and 80–1500 Hz, respectively.

## 3. Results and Discussion

**Figure 1:**
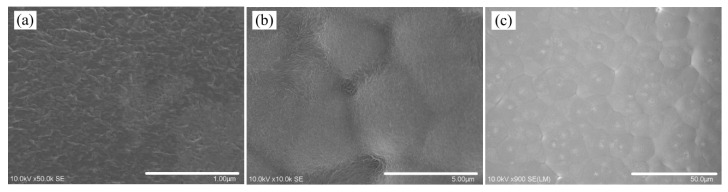
The STEM micrograph of the PVDF films. (**a**) R1, (**b**) R2, (**c**) R3.

By employing the STEM, the morphologies of the PVDF films were obtained, as shown in the Supplementary Materials. It can be seen that the microstructure morphologies and corresponding distribution configurations of samples with a thickness of 5 μm are similar to those of sample groups with thicknesses of 10 μm and 15 μm, respectively. For the sake of clarity, only the morphologies of film samples R1, R2, and R3 are presented in Figure 1, and different scanning magnitudes and graphic scales were adopted during the STEM observation.

As shown in Figure 1, the polygonal morphologies of all the PVDF films are grain-like. By analyzing these morphology configurations with ImageJ Software (v1.8.0), the average grain size (GS) and aspect ratio (*h*/GS) of each film sample are obtained as listed in Table 2. Adopting the classification method for ferroelectric ceramic materials, samples R1, S1, and T1 are nano-grained (GS < 100 nm), R2, S2 and T2 are micro-grained (1 μm < GS < 10 μm), and R3, S3 and T3 are coarse-grained (GS > 10 μm). Considering that the film thickness of the samples is 5 μm, 10 μm and 15 μm, respectively, the samples are of nano-grained when *h*/GS > 50, fine-grained when 5 < *h*/GS < 50, micro-grained when 0.5 < *h*/GS < 5, and coarse-grained when *h*/GS < 0.5. For the sake of clarity, in the present study, the phase transition behavior and dielectric and ferroelectric properties of nano-grained, micro-grained, and coarse-grained samples are shown and compared in groups.

**Figure 2:**
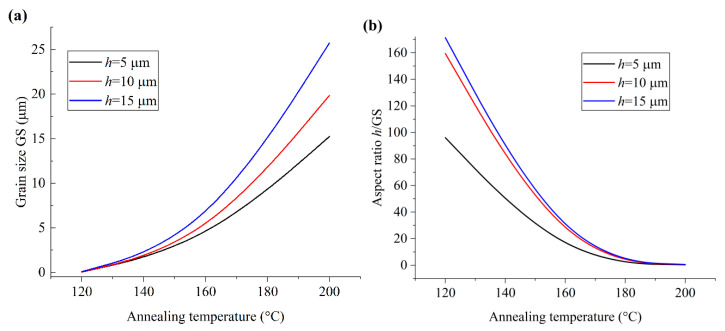
The variation in grain size and aspect ratio with the annealing temperature. (**a**) Grain size; (**b**) aspect ratio.

To analyze the influence of film thickness and corresponding size effect on the microstructure morphology of PVDF films, the variations in GS and *h*/GS of the films with the annealing temperature are illustrated in Figure 2. It can be seen that, with certain annealing temperature, both GS and *h*/GS increase with the film thickness. Hence, with respect to the microstructure configuration, the extrinsic effect corresponding to film thickness is similar to that of the grain boundaries, which is consistent with the phenomena reported in the previous works [8]. The aspect ratio (*h*/GS) is more effective to reflect the size effect on the microstructure and dielectric and ferroelectric properties of the material [6,9] and is adopted in the present analysis.

Meanwhile, as illustrated in Figure 2b, *h*/GS decreases with increasing annealing temperature. Note that the increasing grain size with the annealing temperature is the result of the enhanced molecular chain mobility and suppressed intra-molecular interaction [10]. Hence, the decrease in *h*/GS reflects the increase in the size effect on the microstructure morphology, and the increasing size effect enhances the mobility of molecular chains. Moreover, compared with the case of PVDF films with glass substrate, as illustrated in the previous work [5], it can be seen that the electrical boundary condition has great influence on the microstructure morphology of PVDF films, and the size effect of the PVDF film with a semi-conducting or conducting substrate is more positive to enhance the mobility of the molecular chains.

**Figure 3:**
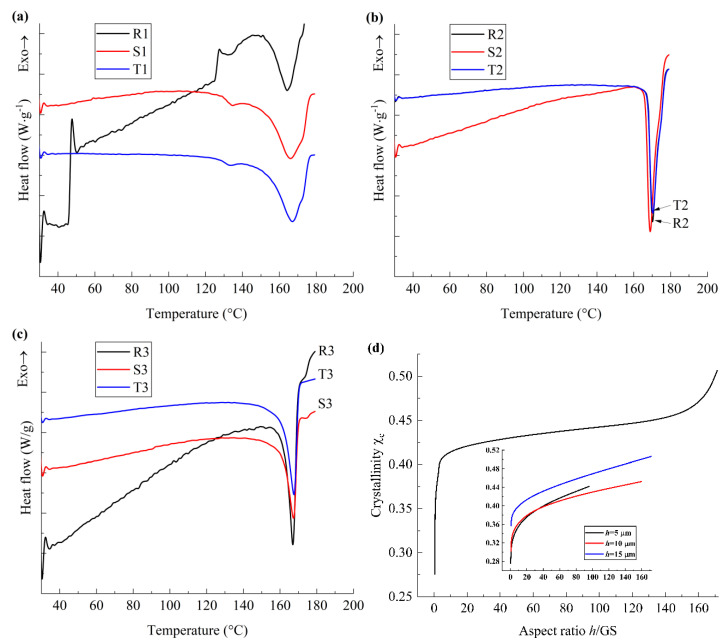
DSC first heating curves for PVDF films. (**a**) DSC thermograms for nano-grained films; (**b**) DSC thermograms for micro-grained films; (**c**) DSC thermograms for coarse-grained films; (**d**) the variation in the crystallinity with aspect ratio.

In order to investigate the phase transformation and crystallization behaviors of the PVDF films, DSC measurement was performed and the thermodynamic parameters of PVDF films were extracted, as shown in Figure 3. As illustrated in Figure 3a–c, for all film samples, one endothermic peak appears at about 164 °C, which corresponds to the melting temperature (*T*_m_) of the films and is smaller than that of the bulk material as reported in previous works [11], which is ascribed to the surface effect of the films [12,13]. At the same time, the endothermic and exothermic events occur successively at around 26–38 °C, and the enthalpy variation decreases with the increasing *h*/GS. Note that the molecular motion of polymer materials originates at the grain boundaries between the crystalline and amorphous phases [14,15]; we may ascribe the variation in the enthalpy around 26 °C and 38 °C to the transformation of molecular chains in the vicinity of the grain boundaries, which is ascribed to the phase transition from a glass phase to a rubbery state [16], and the critical energy for this phase transition is enhanced with the decreasing *h*/GS.

For the nano-grained samples, as illustrated in Figure 3a, the endothermic event is obvious at around 133 °C and the enthalpy variation increases with the decreasing *h*/GS, which is related to the ferroelectric−paraelectric phase transition of PVDF films [14]. Note that the interfacial or spatial charges accumulate in the vicinity of grain boundaries and result in the polarization of the molecular chains [17], while the charge density at the grain boundaries increases with the decreasing *h*/GS. Hence, the critical energy of ferroelectric-paraelectric phase transition is greatly influenced by the aspect ratio of the nano-grained PVDF films due to the density of the interfacial charges. For sample R1, the DSC curve shows sharp changes at around 38–53 °C and 122–130 °C, which indicate the first-order phase transition from glass phase to a rubbery state and the rubbery state to a viscous state, respectively, and are denoted as *T*_g_u_ [18] and *T*_f_ [19]. In other words, the first-order martensitic transition occurs in the vicinity of the grain boundaries for nano-grained PVDF films with small *h*/GS. In addition, a slight entropy vibration was also observed at around 60 °C for sample S1, which reflects the second-order phase transition in the vicinity of the grain boundaries. Hence, for nano-grained PVDF films, the influence of interfacial polarization on the phase transition behavior is more pronounced than that of the mechanical constraint, and the effect of interfacial polarization can be neglected when *h*/GS > 160.

For micro-grained film samples, as illustrated in Figure 3b, when 38 °C < *T* < 125 °C, the heat flow curves increase almost linearly with the temperature and the local vibration is not obvious, while an inflection point appears at around 125 °C; i.e., second-order phase transition takes place at around 125 °C. Hence, for the micro-grained film samples, the material is in a rubbery state when 38 °C < *T* < 125 °C. At the same time, the heat flow curves of samples R2 and T2 almost coincide, and the variation rate of the heat flow curve for sample S2 is much larger than that for samples R2 and T2. It should be noted that the larger variation rate of the heat flow curve reflects the larger specific heat capacity (*C*_p_) and larger cohesive energy of the polymer materials. Considering that the cohesive energy of the β phase of PVDF is much larger than that of α- and γ-phases [20], it may be inferred that the largest fraction ratio of ordered crystalline phases, whose lattice symmetry is similar to that of the polar β phase, is formed when *h*/GS is about 3, due to the balance of the extrinsic effect and the intrinsic effect, which is ascribed to the interfacial polarization and the van der Waals interaction, respectively.

In a similar manner, the exothermic and endothermic behaviors of the coarse-grained films can be analyzed, as illustrated in Figure 3c. Neither the martensitic phase transition nor the ferroelectric phase transition can be observed, while the variation rate of the heat flow curve of R3 is much larger than that of S3 and T3. Note that *h*/GS of coarse-grained films is one order smaller than that of micro-grained films; the effective lattice spacing increases due to the increasing size effect, as reported in the work of Ikeda and Suzuki [21]. Hence, for coarse-grained films, with the decreasing *h*/GS, the fraction ratio of polar phases increases, and the effect of interfacial polarization corresponding to the size effect is larger than that of mechanical constraint. Moreover, when *h*/GS is about 0.3, the intra-molecular interaction and inter-molecular interaction of the material are generally balanced, which results in the largest fraction ratio of the phases with ordered lattice symmetry.

The crystallinity of the PVDF films was extracted from the DSC curves, as shown in Figure 3d, while the crystallinity of the material was denoted as *χ*_c_. It can be seen that *χ*_c_ increases with the increasing *h*/GS, while the variation ratio of *χ*_c_ to *h*/GS for coarse-grained films is the largest, and that for nano-grained films is the smallest. Note that the intra-molecular potential of the molecular chains affects the crystallization behavior of the PVDF films more obviously than the inter-molecular potential, as reported in the work of Hasegawa et al. [22]. Hence, for coarse-grained PVDF films, the size effect on the configuration of molecular chains and corresponding cohesive energy for coarse-grained films is pronounced, while that for micro-grained films is much smaller, and that for nano-grained films is the smallest. These phenomena are consistent with the results obtained from Figure 3a–c.

**Figure 4:**
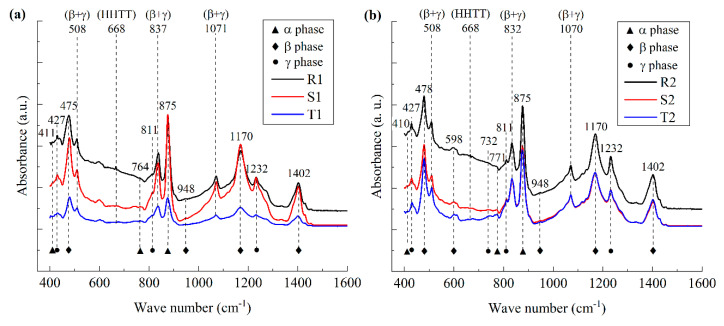

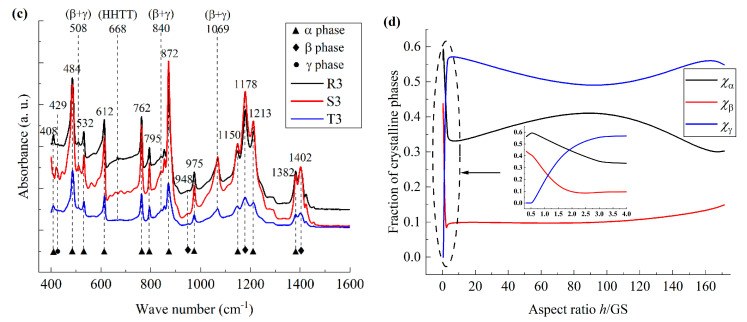
FT-IR spectrum of PVDF films. (**a**) FT-IR spectrum of nano-grained films; (**b**) FT-IR spectrum of micro-grained films; (**c**) FT-IR spectrum of coarse-grained films; (**d**) the variation in the fraction of crystalline phases with aspect ratio.

**Figure 5:**
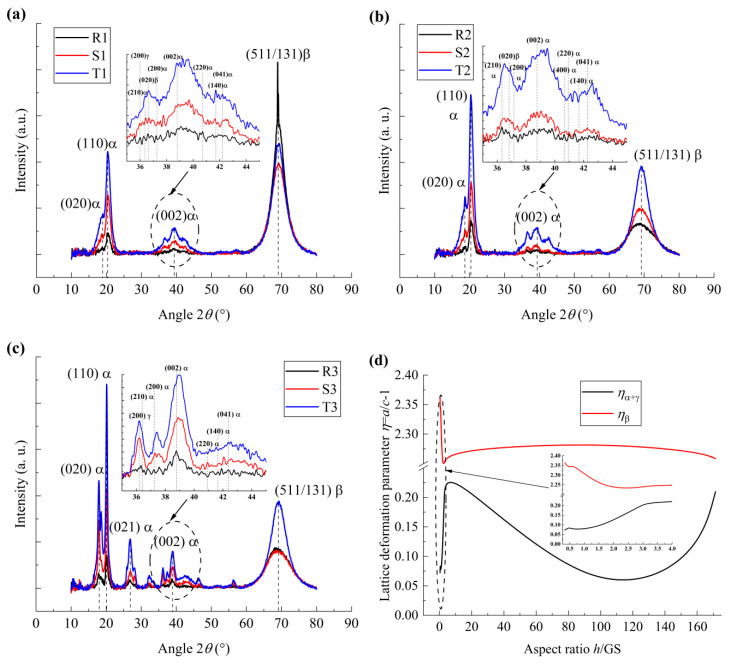
XRD spectrum of PVDF films. (**a**) XRD spectrum of nano-grained films; (**b**) XRD spectrum of micro-grained films; (**c**) XRD spectrum of coarse-grained films; (**d**) the variation in the lattice deformation parameters with aspect ratio.

To further analyze the size effect on the configuration of the molecular chains of the PVDF films, the crystal structures of the PVDF films were analyzed by FT-IR and XRD, as illustrated in Figure 4 and Figure 5, respectively.

From Figure 4a–c, it can be seen that, for all the PVDF film samples, HHTT defects appear at the wave number around 668 cm^−1^, which is consistent with the previous works reported [15]. Meanwhile, polar phases with mixed β- and γ-phases were observed at wave numbers around 508 cm^−1^, 837 cm^−1^, and 1071 cm^−1^. Hence, the polarization behavior of the ferroelectric polymers is mainly induced by the interfacial polarization in the vicinity of the grain boundaries and the polarization switching within the crystalline grains.

By employing the method employed in previous works [23], the fraction of each crystalline phase is extracted from the FT-IR spectroscopy curves and illustrated in Figure 4d, and the fraction ratios of α-, β-, and γ-phases are denoted as *χ*_α_, *χ*_β_, and *χ*_γ_, respectively. It can be seen that, for the nano-grained films, with the increasing *h*/GS, *χ*_α_ decreases while *χ*_β_ and *χ*_γ_ increase. The variation rate of *χ*_γ_ to *h*/GS is larger than that of *χ*_β_. Hence, with the increasing size effect, the depolarization field increases mainly in the polar γ phase, and the influence of the depolarization field corresponding to the local molecular transformation is larger than that of interfacial polarization. On the contrary, for micro-grained films, with the increasing *h*/GS, *χ*_γ_ increases while both *χ*_α_ and *χ*_β_ decrease. Hence, both the polarization and depolarization behaviors coexist in the material; i.e., both the influences of interfacial polarization and the mechanical constraint in the vicinity of the grain boundaries greatly affect the configuration of the molecular chains. Meanwhile, for coarse-grained films, with the increase in *h*/GS, *χ*_β_ decreases and *χ*_α_ increases, while *χ*_γ_ remains relatively constant, i.e., the size effect results in the ordering of molecular motion, and the interfacial polarization effect is larger than that of the depolarization effect. Collectively, the mechanism mentioned above for the polarization behavior of PVDF films is consistent with that for the phase transition behavior of the material obtained from the DSC measurement, as shown in Figure 3.

To further analyze the microstructure configuration of the PVDF films, the XRD measurement was taken, as illustrated in Figure 5. It is noteworthy that the PVDF films containing the γ phase always contains a large amount of the α phase, as reported in previous works [24], while both the lattice parameters *a* and *b* of α- and γ-phases are similar. In the present study, the lattice deformation of α- and γ-phases was not explored in detail in the analysis of the XRD spectrum. Both the peaks corresponding to α- and γ-phases are denoted as α peaks, as shown in Figure 5a–c. In addition, the peak vibration is obvious when the scanning angle 2*θ* is about 40° for all the film samples, as shown in the insert in Figure 5a–c. However, the α phase forms the majority and the fraction ratio of β and γ phases can be neglected. Hence, it is denoted as α phase at 2*θ* ≈ 40°.

The normalized lattice deformation parameter (*η* = *a*/*c* − 1) is extracted based on the XRD spectroscopy curves, as shown in Figure 5d, and the lattice deformation parameter of the α- or γ-phase and β phase was denoted as *η*_α+γ_ and *η*_β_, respectively. It can be seen that, for nano-grained films, *η*_β_ and *η*_α+γ_ decrease and increase with the increasing *h*/GS, respectively. And the variation rate of *η*_β_ to *h*/GS is smaller than that of *η*_α+γ_. Note that both *χ*_β_ and *χ*_γ_ increase with the increasing *h*/GS for nano-grained samples, as illustrated in Figure 4d, which is inconsistent with the variation tendency of *η*_β_ and *η*_α+γ_. Considering that the first-order phase transition takes place in nano-grained films, as illustrated in Figure 3a, it may be inferred that, with the increasing size effect, IAF→OAF phase transformation takes place, while the lattice parameters and resonant vibration energy of the OAF phase are similar to those of the β phase and α phase, respectively. Hence, with the increasing size effect, phase transformations (β→α, γ→α, and IAF→OAF) take place simultaneously, and the formation of OAF is mainly ascribed to the effects of interfacial polarization and electrostriction.

On the contrary, for micro-grained samples and coarse-grained samples, with the increasing *h*/GS, *η*_β_ and *η*_α+γ_ decrease and increase, respectively, while both the variation rates of *η*_β_ and *η*_α+γ_ to *h*/GS for coarse-grained samples are larger than those for micro-grained samples. Hence, the phase transformations (γ→β and γ→α) take place simultaneously for micro-grained PVDF films due to the increasing size effect, whereas the main phase transformation is in the form of β→α for coarse-grained PVDF films. These phenomena are consistent with the observation from the FT-IR measurement mentioned above.

**Figure 6:**
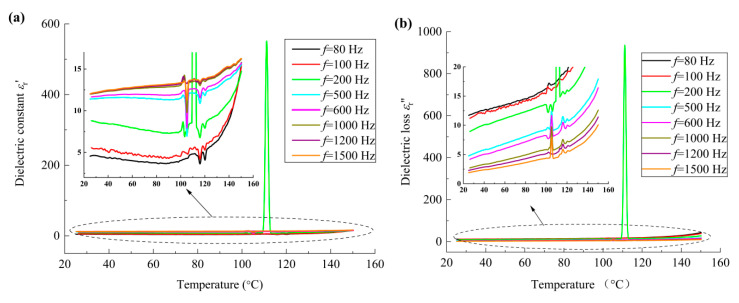
Temperature dependence of dielectric constant and dielectric loss of nano-grained PVDF films at various frequencies: (**a**) dielectric constant; (**b**) dielectric loss.

Moreover, to analyze the motion of the molecular chains and the corresponding dielectric properties of the material, the variation in the dielectric constant (*ε*_r_′) and dielectric loss (*ε*_r_″) with respect to temperature were measured. Note that, in the previous work of the authors [5], it was found that both *ε*_r_′ and *ε*_r_″ vary nonlinearly with the frequency when the frequency is smaller than 1500 Hz, while *ε*_r_′ and *ε*_r_″ remain relatively constant when the frequency is larger than 1500 Hz. Meanwhile, both the variation tendency of the dielectric constant and dielectric loss with respect to the frequency for all nano-grained, micro-grained and coarse-grained film samples are similar. For the sake of clarity, the frequency range of 80 Hz to 1500 Hz was adopted in the present study. And the dielectric spectrum of sample R1 at various frequencies is shown in Figure 6. It can be seen that the variation tendency of both dielectric constant and dielectric loss curves with frequency has three distinct regions, i.e., *f* ≤ 100 Hz, 100 Hz < *f* < 500 Hz, and *f* ≥ 500 Hz, which are mainly ascribed to the effects of interfacial charges, mechanical deformation, and dipolar moments, respectively. The dielectric constant reaches the minimum at around 90 °C, and this phenomenon is ascribed to the phase transition at the interfaces between the crystalline phase and amorphous phase. At around 100–120 °C, the sharp vibration of the dielectric constant and dielectric loss takes place, which is ascribed to the phase transition of the rubbery state to viscous phase of the material. And the sudden change in both dielectric constant and dielectric loss curves at *f* ≈ 200 Hz and *T* ≈ 110 °C indicates the obvious molecular transformation corresponding to the electrostriction effect of the material.

**Figure 7:**
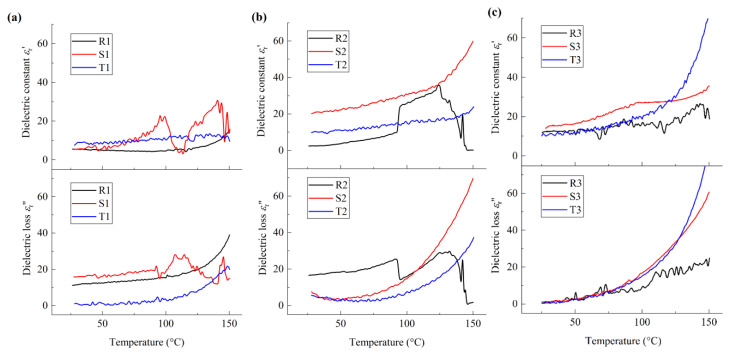
Temperature dependence of dielectric constant and dielectric loss of PVDF films at *f* = 100 Hz: (**a**) nano-grained films; (**b**) micro-grained films; (**c**) coarse–grained films.

**Figure 8:**
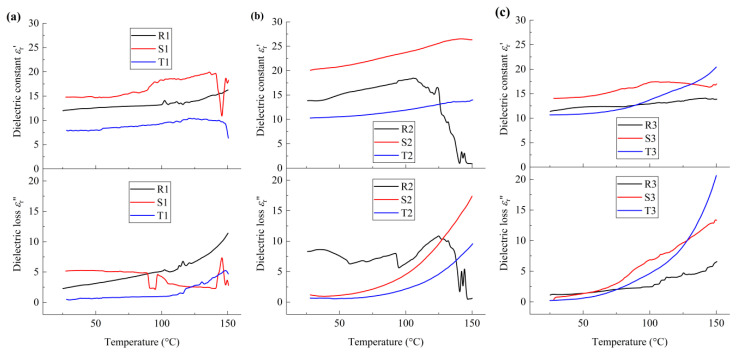
Temperature dependence of dielectric constant and dielectric loss of PVDF films at *f* = 1200 Hz: (**a**) nano-grained films; (**b**) micro-grained films; (**c**) coarse−grained films.

To analyze the influences of interfacial charges and dipolar moments on the deformation of molecular chains in detail, the temperature dependence of the dielectric constant and dielectric loss at *f* = 100 Hz and *f* = 1200 Hz for all PVDF film samples were compared and are illustrated in Figure 7 and Figure 8, respectively. It should be noted that silicon substrates were adopted in the present experiment, space charges exist in the films, and the motion of space charges can be easily influenced by the temperature. Hence, the dielectric thermo-grams of the film samples at frequency *f* = 100 Hz show obvious local vibration, as shown in Figure 7.

From Figure 7a, it can be seen that, at around 34 °C, local molecular motion, denoted as *α*_a_ relaxation [25], is observed for all nano-grained samples. With reference to Figure 3a and Figure 5a, it can be seen that the phase transformation in the vicinity of the grain boundaries initiates from the amorphous phase. With increasing temperature, both *ε*_r_′ and *ε*_r_″ increase nonlinearly for sample R1, while local vibration occurs at around 100 °C, which is ascribed to the local molecular relaxation in the vicinity of the grain boundaries and denoted as *α*_c_ relaxation [26]. Similarly, *α*_c_ relaxation is observed at around 114 °C for sample S1. Meanwhile, the obvious vibration of *ε*_r_′ of sample T1 can be neglected when *T* < 100 °C and *α*_c_ relaxation is observed at around 125 °C. Hence, for nano-grained PVDF films, the molecular motion occurs in both amorphous phase and crystalline phase, and the temperature for *α*_c_ relaxation increases with *h*/GS. With reference to Figure 3a and Figure 5d, the above mentioned phenomena may be ascribed to the effects of interfacial polarization and related electrostriction in the vicinity of the grain boundaries, and the local molecular rotation of the polar crystalline phase is weakened correspondingly. The formation of the OAF phase may be due to the balance of the electrostriction effect corresponding to the interfacial polarization and the mechanical constraint in the vicinity of the grain boundaries.

From Figure 7b, it can be seen that, with the increase in temperature, a sudden increase in *ε*_r_′ and decrease in *ε*_r_″ are observed at around 94 °C for sample R2, which is ascribed to the first-order IAF-OAF phase transition. Note that *h*/GS of micro-grained films is about one order smaller than that of nano-grained films; the size effect of micro-grained films on the microstructure configuration increases accordingly, which results in an increase in the effective lattice spacing and the density of interfacial charge. Hence, the phase transition of micro-grained PVDF films is a result of the combined effects corresponding to mechanical constraint and interfacial polarization. Meanwhile, for samples S2 and T2, *ε*_r_′ increases nonlinearly with the increasing temperature and *ε*_r_″ decreases first and then increases, reaching the minimum at around 53 °C. Note that no obvious variation in the heat entropy is observed at around 53 °C for these film samples, as shown in Figure 3b. Namely, the above-mentioned phenomenon can be ascribed to the local molecular motion of the constrained amorphous phase in the vicinity of the grain boundaries, i.e., *T*_g_u_ ≈ 53 °C, which can induce the phase transition between the γ phase and β phase in the crystalline phases, as illustrated in the insert of Figure 4d. In addition, one inflection point appears at around 122 °C for sample S2, and the variation ratio of the dielectric constant of sample S2 is larger than that of sample T2. Namely, the second-order phase transition of sample S2 and T2 is similar to that of relaxor ferroelectrics, and the inter-molecular interaction of sample S2 is larger than that of sample T2. Hence, for micro-grained PVDF films, with the increasing *h*/GS, the inter-molecular interaction decreases, and the fraction ratio of polar nano-regions (PNRs) increases as a result of the increasing depolarization field and decreasing interfacial polarization. With reference to Figure 4d and Figure 5d, it can be seen that the interaction between the amorphous phase and the crystalline phases induces the formation of PNRs in the crystalline phase, which is consistent with the previous work [7]. In addition, comparing R2 and T2, *ε*_r_′ of sample S2 is the largest when 25 °C < *T* < 94 °C. Hence, at the service temperature range, when *h*/GS is about 3, the fraction ratio of the polar phase is maximal and it behaves in accordance with relaxor ferroelectrics as a result of the balance between the depolarization field corresponding to the molecular motion and that of the interfacial polarization.

Similarly, the dielectric properties of coarse-grained PVDF films can be analyzed, as illustrated in Figure 7c. It can be seen that the first-order phase transition is not observed for all samples. For samples R3 and T3, when the temperature is lower than 100 °C, the curves of both *ε*_r_′ and *ε*_r_″ coincide, and the variation rate of *ε*_r_′ with respect to temperature is smaller than that of *ε*_r_″, which means that the configuration of the molecular chains for samples R3 and T3 are similar, and the local molecular chain motion occurs in the nonpolar α crystalline phase. In contrast, for sample S3, the nonlinear increase in both *ε*_r_′ and *ε*_r_″ is observed and one inflection point appears at the dielectric constant curve at around 100 °C, which is denoted as *α*_1_ relaxation [27]. Hence, the motion of molecular chains of coarse-grained PVDF films is mainly of local relaxation in the amorphous phase and nonpolar α crystalline phase, and the second-order phase transition of sample S3 is ascribed to the diffusional phase transition from a rubbery state to the amorphous phase. It should be noted that the motion of the molecular chains of both the nonpolar α phase and the amorphous phase is dominated by the van der Waal interaction among the molecular chains. With reference to Figure 3c, Figure 4d and Figure 5d, it can be seen that, for coarse-grained PVDF films, the molecular motion is mainly dominated by the intra-molecular van der Waal interaction, while the molecular configuration can be affected by the interfacial charges in the vicinity of grain boundaries. The second-order phase transition is obvious when *h*/GS is about 0.5, while the effects of the inter-molecular and intra-molecular interactions among the molecular chains are balanced.

In a similar manner, the dielectric behavior of the films at *f* = 1200 Hz can be analyzed, as shown in Figure 8. From Figure 8a, it can be seen that, for sample R1, local molecular motion in the nonpolar crystalline phase and polar crystalline phase was observed at 37 °C and 116 °C, which is ascribed to α_1_ relaxation and α_2_ relaxation, respectively. For sample S1, at around 90 °C, a smooth increase and sudden drop in *ε*_r_′ and *ε*_r_″ were observed, respectively, which is ascribed to the molecular motion in the amorphous phase and the first-order phase transition between the rubbery state and viscous state in the vicinity of the grain boundaries. Meanwhile, a sharp decrease and increase in *ε*_r_′ and *ε*_r_″ are observed, respectively, at around 145 °C, which is ascribed to the molecular motion in the crystalline phase and the ferroelectric–paraelectric phase transition of the material. Moreover, compared with the cases shown in Figure 7a, it can be seen that the degree of phase transition induced by dipolar switching is smaller than that of the interfacial charges. Hence, the interfacial charge has a larger influence on the dielectric properties and microstructure of the PVDF films, which is consistent with the analysis of the influence of electrical boundary condition on the microstructure morphology, as mentioned above.

At the same time, for sample T1, the variation tendency of *ε*_r_′ and *ε*_r_″ is generally similar to that of sample R1, while *α*_1_ relaxation and *α*_2_ relaxation were observed at 40 °C and 110 °C, respectively. At the service temperature range of PVDF films, both *ε*_r_′ and *ε*_r_″ of sample S1 are larger than those of sample R1 and T1, while *ε*_r_′ and *ε*_r_″ of sample T1 are the smallest. Hence, for nano-grained PVDF films, with the increasing *h*/GS, the degree of the polarization increases first and then decreases, due to both effects corresponding to the mechanical constraint and the interfacial polarization. The fraction ratio of polar phases is the maximum when *h*/GS is about 160, due to a balance between the effects of the interfacial polarization and the mechanical constraint.

From Figure 8b, it can be seen that the local molecular motion corresponding to relaxor ferroelectric phase transition was observed for sample R2 at around 58 °C, 93 °C and 125 °C, respectively, which correspond to molecular relaxation in the OAF phase and the interfaces of the crystalline phase, and the ferroelectric–paraelectric phase transition. On the contrary, for samples S2 and T2, both *ε*_r_′ and *ε*_r_″ increase nonlinearly with the increasing temperature. When 25 °C < *T* < 93 °C, *ε*_r_′ of sample S2 is larger than that of samples R2 and T2, and *ε*_r_″ of sample R2 is larger than that of samples S2 and T2. Hence, the ferroelectric behavior of micro-grained PVDF films is similar to that of relaxor ferroelectrics, and the polar nano-regions are formed due to the interaction between the OAF phase and crystalline phase. The influence of dipolar moments on the dielectric and ferroelectric properties decreases with the increasing *h*/GS due to the decreasing size effect and decreasing density of interfacial charges in the vicinity of the grain boundaries. When *h*/GS is about 3, the polarization field and the van der Waals interaction are balanced, and the fraction ratio of polar crystalline phase is the maximum.

Similarly, no obvious first-order phase transition is observed for the coarse-grained films, as shown in Figure 8c, and the variation rate of *ε*_r_″ with respect to temperature is much larger than that of *ε*_r_′. In addition, compared with the dielectric behaviors of R3 and T3, the *ε*_r_′ and *ε*_r_″ of sample S3 are the largest when 25 °C < *T* < 125 °C. Hence, for coarse-grained PVDF films, the intra-molecular van der Waals interaction plays a major role in the dielectric properties of the material, while the molecular configuration can be affected by the size effect of the grain boundaries. When *h*/GS is about 0.5, the fraction ratio of the polar crystalline phase is the maximum, as a result of the balance between the intra-molecular interaction and the inter-molecular interaction among the molecular chains.

**Figure 9:**
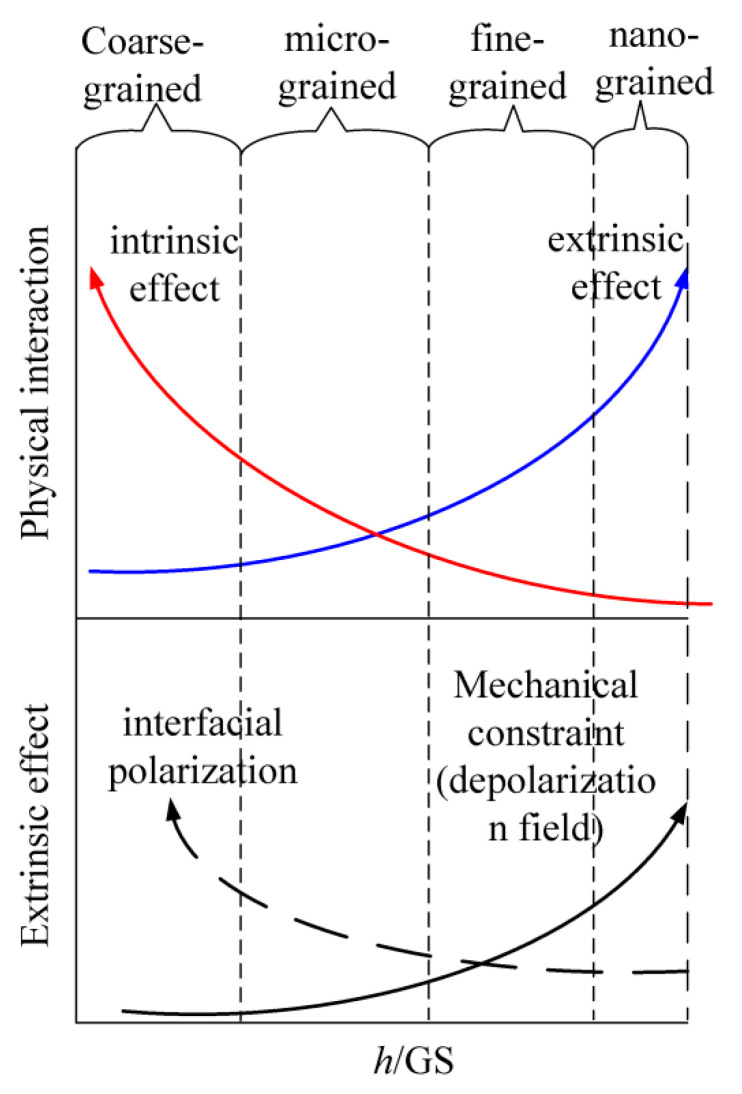
Schematic of the variation tendency of intrinsic effect and extrinsic effect with the aspect ratio.

Above all, the size effect on the dielectric properties of the PVDF films is the result of both the extrinsic effect and intrinsic effect, which can be generally illustrated as shown Figure 9. The electrical extrinsic effect mainly corresponds to the interfacial charges or space charge in the vicinity of the grain boundaries; the mechanical extrinsic effect mainly corresponds to the mechanical stress and the depolarization field induced; and the intrinsic effect mainly corresponds to the intra-molecular van der Waal interaction. Note that, based on the results of the present work, the mechanism of the size effect for fine-grained films is proposed as shown in Figure 9, and it will be investigated in detail and reported in the follow-up work of the authors.

## 4. Conclusions

In this study, by employing multi-scale observation methods, the size effect on the dielectric properties and phase transition behavior of PVDF ferroelectric polymer films was analyzed, while the influences of both the film surface and grain boundaries were taken into account. It was found that the oriented amorphous fraction phase is prone to form in the vicinity of the interfacial layer of the nano-grained films. The lattice parameters of the OAF phase are similar to those of the β phase, and the resonant vibration energy of the OAF phase is similar to that of the α phase. Polar nano-regions are prone to form in the vicinity of the interfacial layer of the micro-grained films.

For nano-grained ferroelectric polymer films, the first-order IAF↔OAF phase transition takes place, where the interfacial polarization and electrostriction effect play a major role, and the materials reach their maximal dielectric constant and minimal dielectric loss when *h*/GS is about 160 at the service temperature of the material. On the contrary, for micro-grained ferroelectric polymer films, both the intrinsic effect and extrinsic effect play a major role in the behavior of the dielectric properties and ferroelectric properties, and the polar nano-regions are prone to originate in the vicinity of the grain boundaries. The maximal fraction of polar phases can be obtained when *h*/GS is about 3. For coarse-grained ferroelectric polymer films, the intrinsic interaction among the molecular chains plays a major role in the dielectric properties and ferroelectric properties, while the interfacial charges can influence the configuration of the molecular chains, and the maximal fraction of the polar phase can be obtained when *h*/GS is about 0.5.

These phenomena are important for the design and manufacture of ferroelectric polymer devices. The dielectric and ferroelectric properties of nano-grained ferroelectric polymer films can be effectively adjusted by copolymerization techniques. In contrast, those of the micro-grained ferroelectric polymers can be optimized by controlling the fraction ratio of polar nano-regions. Meanwhile, the ferroelectric properties of coarse-grained ferroelectric polymer films can be adjusted by the doping technique and the application of electrical loading.

## Figures and Tables

**Table 1:** Spin-coating rates and annealing conditions of PVDF films.

Sample No.	Film Thickness (μm)	Spin-Coating Rate (rmp)		Annealing Temperature (°C)
		Lower Spin Rate	Higher Spin Rate	
R1	5	700 rpm for 40 s	4000 rpm for 20 s	120
R2	5	700 rpm for 40 s	4000 rpm for 20 s	160
R3	5	700 rpm for 40 s	4000 rpm for 20 s	200
S1	10	600 rpm for 45 s	1600 rpm for 20 s	120
S2	10	600 rpm for 45 s	1600 rpm for 20 s	160
S3	10	600 rpm for 45 s	1600 rpm for 20 s	200
T1	15	500 rpm for 25 s	1000 rpm for 25 s	120
T2	15	500 rpm for 25 s	1000 rpm for 25 s	160
T3	15	500 rpm for 25 s	1000 rpm for 25 s	200

**Table 2:** The average grain size and aspect ratio of PVDF films.

Sample No.	GS (μm)	*h*/GS
R1	0.05207	96.0246
R2	3.14	1.5924
R3	15.24	0.3281
S1	0.06273	159.4134
S2	3.31	3.0211
S3	19.86	0.5035
T1	0.08753	171.3708
T2	3.91	3.8358
T3	25.73	0.5830

## Data Availability

The original contributions presented in this study are included in the article. Further inquiries can be directed to the corresponding author.

## Supplementary Materials

The following supporting information can be downloaded at https://www.mdpi.com/article/10.3390/polym17091286/s1: Figure S1. The STEM micrograph of the PVDF films. (a) R1, (b) R2, (c) R3, (d) S1, (e) S2, (f) S3, (g) T1, (h) T2, (i) T3.
