# Supplementary material for: The Size Effect on the Phase Transition and Dielectric Properties of Poly(vinylidene Fluoride) Ferroelectric Polymers
Source: Polymers (Basel). 2025 May 7;17(9):1286. doi: 10.3390/polym17091286 (PMC12074403; doi:10.3390/polym17091286)
Supplement: Supplementary file 1 [file polymers-17-01286-s001.zip › polymers-3592866-supplementary.pdf]

Supplementary Material for

The size effect on the phase transition and dielectric properties of poly(vinylidene fluoride) ferroelectric polymers

Xiaofang Zhao *, Min Yu and Xining Zhang

School of Mathematics, Statistics and Mechanics, Beijing University of Technology, Beijing 100124, China

* Correspondence: xfzhao@bjut.edu.cn

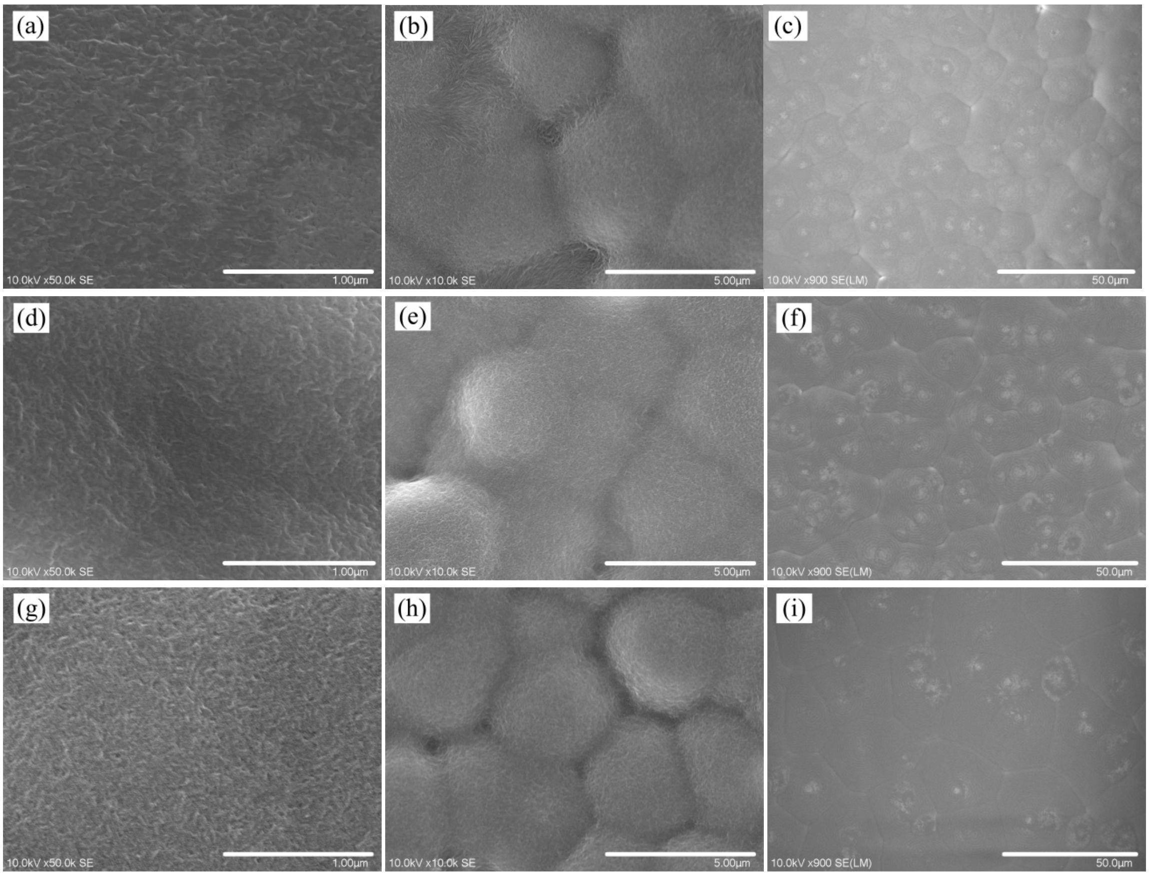

Figure S1. The STEM micrograph of the PVDF films.
(a) R1, **(b)** R2, **(c)** R3, **(d)** S1, **(e)** S2, **(f)** S3, **(g)** T1, **(h)** T2, **(i)** T3.
